# Use and effectiveness of the Individual Development Plan among postdoctoral researchers: findings from a cross-sectional study

**DOI:** 10.12688/f1000research.15610.2

**Published:** 2018-10-25

**Authors:** Nathan L. Vanderford, Teresa M. Evans, L. Todd Weiss, Lindsay Bira, Jazmin Beltran-Gastelum

**Affiliations:** 1 Department of Toxicology & Cancer Biology, University of Kentucky, College of Medicine, 800 Rose Street, KY, USA; 2 Markey Cancer Center, University of Kentucky, 800 Rose Street, KY, USA; 3 Center for Cancer and Metabolism, University of Kentucky, 800 Rose Streer, KY, USA; 4 Department of Pharmacology, University of Texas Health San Antonio, 7703 Floyd Curl Drive, San Antonio, TX, USA; 5 Department of Psychiatry, University of Texas Health San Antonio, 7703 Floyd Curl Drive, San Antonio, TX, USA; 6 Department of Pharmacology and Toxicology, University of Arizona, College of Pharmacy , 1295 N. Martin Ave, Tucson, AZ, USA

**Keywords:** biomedical research, career development, careers in research, career planning, individual development plan, PhD training, postdoctoral researchers, science and technology workforce

## Abstract

The individual development plan (IDP) is a career planning tool that aims to assist PhD trainees in self-assessing skills, exploring career paths, developing short- and long-term career goals, and creating action plans to achieve those goals. The National Institutes of Health and many academic institutions have created policies that mandate completion of the IDP by both graduate students and postdoctoral researchers. Despite these policies, little information exists regarding how widely the tool is used and whether it is useful to the career development of PhD trainees. Herein, we present data from a multi-institutional, online survey on the use and effectiveness of the IDP among a group of 183 postdoctoral researchers. The overall IDP completion rate was 54% and 38% of IDP users reported that the tool was helpful to their career development. Positive relationships with one’s advisor, confidence regarding completing training, trainees’ confidence about their post-training career, and a positive experience with institutional career development resources are associated with respondents’ perception that the IDP is useful for their career development. We suggest that there is a need to further understand the nuanced use and effectiveness of the IDP in order to determine how to execute the use of the tool to maximize trainees’ career development.

## Introduction

The Individual Development Plan (IDP) was first introduced by the U.S. Federation of American Societies for Experimental Biology in 2002, and in 2014 the National Institutes of Health implemented a policy requiring the reporting of the tool’s use by graduate students and postdoctoral researchers in grant progress reports ^1– 3^. Also in 2014, a survey of over 200 postdoctoral researchers found that 19% of respondents used the IDP with 71% of those users finding it valuable ^4^. The IDP has been suggested to be capable of, for example, enhancing the structure of a training environment, facilitating better communication between mentees and mentors, aiding in identifying and pursuing career paths, guiding the identification of skills and knowledge gaps and creating action plans for addressing such gaps ^4, 8– 10^. IDPs are suggested to be a staple career development activity for PhD trainees, especially related to supporting trainees’ preparation for and decisions in navigating a diverse job market ^11^. We suggest, however, that more research is needed to further characterize the use and effectiveness of IDPs in maximizing trainees’ career development. As such, within this report, we present data on the use and effectiveness of the IDP among a group of 183 postdoctoral researchers.

## Methods

These data were collected as part of a broader health and wellbeing online, survey-based study of graduate students and postdoctoral researchers in the spring and early summer of 2016 (March to June). The study was approved by the University of Kentucky (protocol 15-1080-P2H) and University of Texas Health Science Center San Antonio (protocol HSC20160025X) institutional review boards. Respondents read a cover page and anonymously consented to the study by engaging the online survey. The survey was distributed via social media and direct email. To be eligible for this study, respondents had to be current postdoctoral researchers in the life/biological/medical or physical/applied sciences at a U.S. institution. Subjects responded to the IDP questions within the survey using the five-point Likert scale of strongly agree, agree, neutral, disagree and strongly disagree. For data analysis, these items were recoded into three categories: strongly agree and agree became an agree category, disagree and strongly disagree became a disagree category, and neural remained its own category. One-way frequencies were calculated ( Supplementary File 2) and the Pearson chi-square test was used to assess the univariate associations between the survey variables and the outcome “I Find the IDP Process Helpful to my Career Development” only among the respondents who completed an IDP as defined by those unique respondents who agreed with questions 2 or 3 within the survey ( Supplementary File 4). All summaries and statistical analysis were performed in SAS 9.4.

## Results

**Figure 1.**
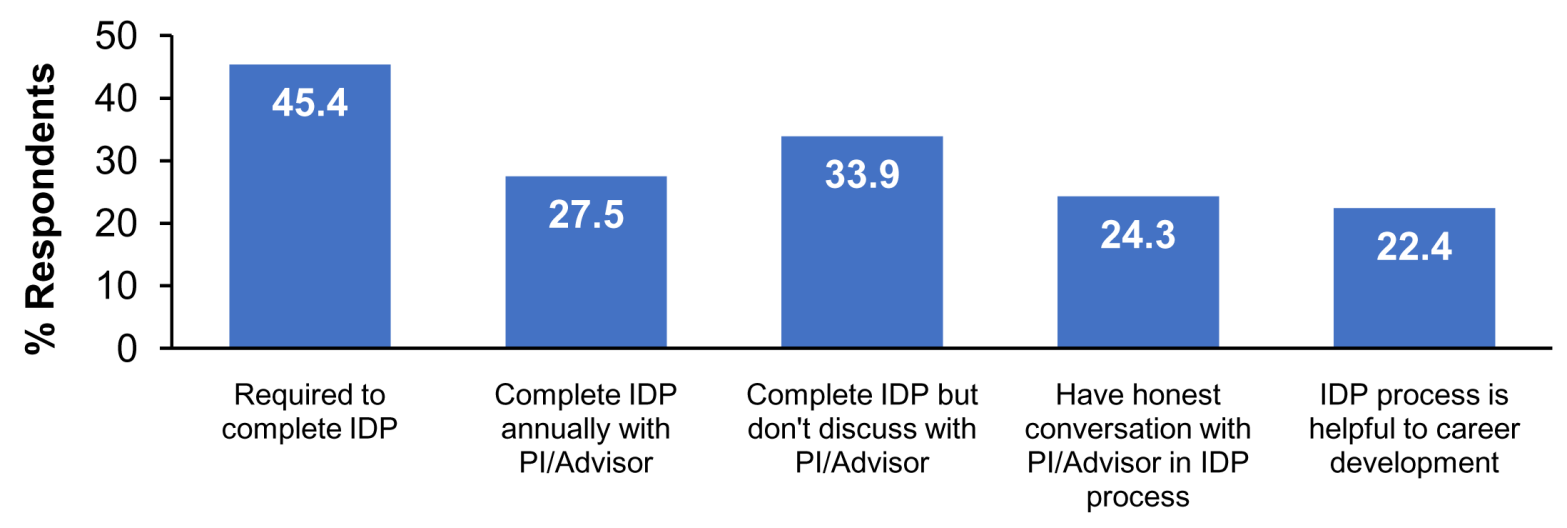
The rates of Individual Development Plan (IDP) use among postdoctoral researchers. Shown here are rates for variables measuring whether respondents are required to complete an IDP, complete an IDP annually with their PI/advisor, complete an IDP but do not discuss it with their PI/advisor, can have an honest conversation with the PI/advisor in context of the IDP, and whether the IDP process is helpful to their career development. One-way frequencies for all other survey variables can be found in Supplementary File 2.

Among 183 total postdoctoral respondents, 45.4% reported being required to complete an IDP, 27.5% reported completing the tool with their PI/advisor, and 33.9% completed the IDP, at some point, without discussing it with their PI/advisor ( Figure 1 and Supplementary File 2). In total, 54.1% of respondents actually completed the IDP with or without their advisor (based on the unique responses to questions 2 and 3 within the survey). Further, 24.3% of all respondents reported being able to have an honest conversation with their PI/advisor in the context of the IDP process ( Figure 1 and Supplementary File 2).

**Figure 2.**
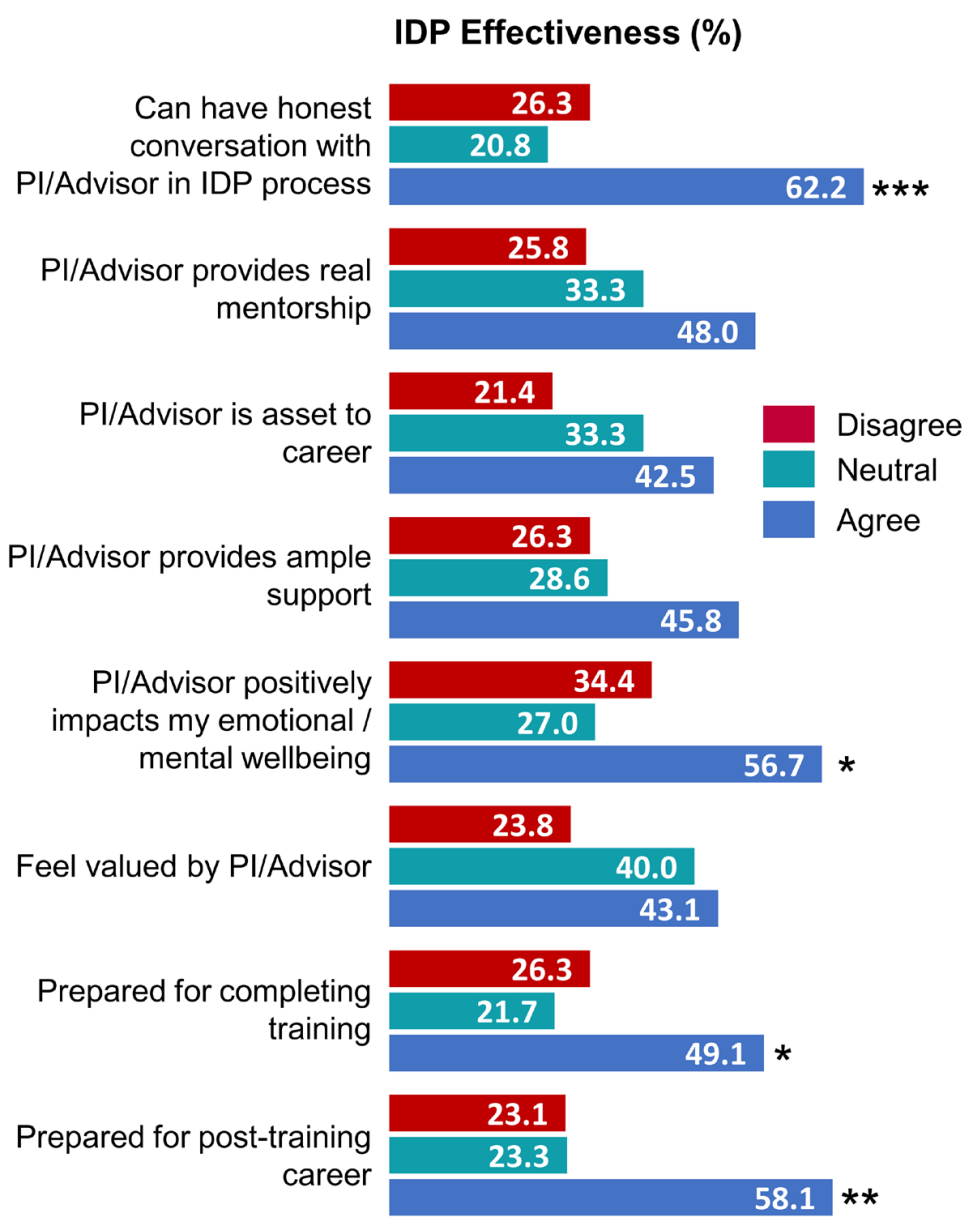
The effectiveness of the Individual Development Plan (IDP). IDP effectiveness was assessed only among the subset of respondents who completed an IDP by determining the univariate associations between the survey variables and the outcome “I Find the IDP Process Helpful to my Career Development.” The Pearson chi-square test was used to measure statistical significance. *** p < 0.001; ** p ≤ 0.01; * p ≤ 0.05.

As a measure of IDP effectiveness, 22.4% of all respondents found the IDP helpful to their career development ( Figure 1 and Supplementary File 2). Among the respondents that completed an IDP, 38.4% found the tool helpful ( Supplementary File 3). As we have recently shown with PhD students ^5^, the effectiveness of the IDP among its users is associated with positive mentorship relationships ( Figure 2 and Supplementary File 3). For example, 62.2% of those respondents who indicated that they could have an honest conversation with their PI/advisor found that the IDP process was helpful to their career versus 26.3% of those who disagreed (p < 0.001). Likewise, 56.7% of those who indicated that their PI/advisor positively impacts their emotional/mental wellbeing versus 34.4% of those who disagreed with this statement found the IDP process to be helpful to their career (p = 0.05). IDP effectiveness was also associated with confidence regarding the completion of training, being prepared for one’s post-training career, and positive interactions with career development resources ( Figure 2 and Supplementary File 3).

Individual Development Plan survey data Columns Q1–Q26 correspond to the questions listed in Supplementary File 4
Copyright: © 2018 Vanderford NL et al. 2018 Data associated with the article are available under the terms of the Creative Commons Zero "No rights reserved" data waiver (CC0 1.0 Public domain dedication).

## Discussion

The IDP is widely touted as a gold standard career development tool even though we know relatively little about its use and effectiveness. Compared to a 2014 study in which 19% of surveyed postdoctoral researchers used the IDP and 71% of users found it valuable ^4^, the current data suggests that there may be a general increase in IDP usage among postdoctoral researchers with 54.1% of respondents in this study indicating that they completed an IDP while its perceived value seems to have decreased to less than 40% of the tool’s users. Additional studies should further understand the overall usage rates and perceived value of the IDP.

In general, the trends presented here for postdoctoral researchers are similar to our recent findings on the use and effectiveness of the IDP in PhD students ^5^, but there are some nuanced differences. For example, compared to the rates in PhD students, the rates of required completion of the IDP among this study’s postdoctoral researchers are lower; the rates of completing the IDP but not discussing it with a PI/advisor are higher; and the rates of reporting that the IDP process is helpful to one’s career development are lower. The correlation of IDP effectiveness and mentorship relationships and use of career development resources are similar between PhD students and postdoctoral researchers. It will be important to conduct additional studies to further delineate differences and similarities in the usage and effectiveness of the IDP between PhD students and postdoctoral researchers.

While this work will add to our understanding of the IDP, there are some limitations to the study including the potential lack of generalizability across all institutions and/or fields of study and potential data/outcome bias. Additionally, this study may not capture all the issues related to the IDP, respondents may not be aware of their institution’s IDP policies, the IDP structure and processes may vary within and between institutions, and the measure of the effectiveness of the IDP herein is subjective and limited. Subjects’ responses may also reflect multiple experiences with the IDP during their training. Given potential differences in study populations and differences in study designs, care should also be taken in comparing this work to other IDP use/effectiveness data. 

Overall, this study demonstrates that IDP use and effectiveness is quite nuanced. Additional research is needed to further understand the use and effectiveness of the IDP. For example, we need a better understanding of all the variations of the IDP used in the community and whether any one variation has advantages over others, whether completing an IDP with or without a mentor leads to varying outcomes, whether the IDP has any influence on career outcomes and much more.

Ultimately, the IDP is likely an effective career development tool in general, but we should better understand how to use it in the most effective way so that we can provide the most positive impact on trainees’ career development. 

## Data availability

The data referenced by this article are under copyright with the following copyright statement: Copyright: © 2018 Vanderford NL et al.

Data associated with the article are available under the terms of the Creative Commons Zero "No rights reserved" data waiver (CC0 1.0 Public domain dedication). 

**Dataset 1. Individual Development Plan survey data. Columns Q1–Q26 correspond to the questions listed in Supplementary File 4. 10.5256/f1000research.15610.d222615^7^**

## Supplementary material

**Supplementary File 1. Self-reported institution of all respondents.**

**Supplementary File 2. One-way frequencies of all respondents, separated by demographic characteristics.**

**Supplementary File 3. Univariate analysis of the survey’s variables and the perception of Individual Development Plan helpfulness among respondents who completed an Individual Development Plan.**

**Supplementary File 4. Example copy of the survey questions relevant to this study.**

## References

[1] Clifford PS: Quality Time with Your Mentor. *Scientist.* 2002;16:59 Reference Source

[2] Hobin JA Fuhrmann CN Lindstaedt B: You Need a Game Plan. *Science.* 2012 10.1126/science.caredit.a1200100

[3] National Institutes of Health: Revised Policy: Descriptions on the Use of Individual Development Plans (IDPs) for Graduate Students and Postdoctoral Researchers Required in Annual Progress Reports beginning October 1, 2014. 2014 Reference Source

[4] Hobin JA Clifford PS Dunn BM: Putting PhDs to work: career planning for today's scientist. *CBE Life Sci Educ.* 2014;13(1):49–53. 10.1187/cbe-13-04-0085 24591503 PMC3940462

[5] Vanderford NL Evans TM Weiss LT: A cross-sectional study of the use and effectiveness of the Individual Development Plan among doctoral students [version 2; referees: 2 approved, 1 approved with reservations]. *F1000Res.* 2018;7:722. 10.12688/f1000research.15154.2 30026933 PMC6039936

[6] Tsai JW Vanderford NL Muindi F: Optimizing the utility of the individual development plan for trainees in the biosciences. *Nat Biotechnol.* 2018;36(6):552–553. 10.1038/nbt.4155 29874221

[7] Vanderford NL Evans TM Weiss LT: Dataset 1 in: Use and effectiveness of the Individual Development Plan among postdoctoral researchers: findings from a cross-sectional study. *F1000Research.* 2018;7: 1132. 10.5256/f1000research.15610.d222615 PMC6240468 30498569

[8] Davis G: Improving the postdoctoral experience: An empirical approach. *Science and engineering careers in the United States: An analysis of markets and employment.* 2009;99–127. Reference Source

[9] Gould J: Career development: A plan for action. *Nature.* 2017;548:489–490. 10.1038/nj7668-489a

[10] Vincent BJ Scholes C Staller MV: Yearly planning meetings: individualized development plans aren't just more paperwork. *Mol Cell.* 2015;58(5):718–721. 10.1016/j.molcel.2015.04.025 26046646

[11] Fuhrmann CN: Enhancing Graduate and Postdoctoral Education To Create a Sustainable Biomedical Workforce. *Hum Gene Ther.* 2016;27(11):871–879. 10.1089/hum.2016.154 27762630 PMC5116696

