# Supplementary material for: Use and effectiveness of the Individual Development Plan among postdoctoral researchers: findings from a cross-sectional study
Source: F1000Res. 2018 Oct 25;7:1132. Originally published 2018 Jul 25. [Version 2] doi: 10.12688/f1000research.15610.2 (PMC6240468; doi:10.12688/f1000research.15610.2)
Supplement: Peer review history

# Peer review: Referee response for version 2

**DOI:** 10.5256/f1000research.18327.r39882

**Published:** 2018-12-04

**Authors:** Tammy Collins

**Affiliations:** 1 Office of Fellows' Career Development, National Institute of Environmental Health Sciences, National Institutes of Health, Research Triangle Park, NC, USA

We thank the authors for their revisions, and feel that the manuscript has been improved and clarified.  The authors addressed most of my initial concerns, but some areas remain to be addressed:

  

1. Reporting values of 24.3% (honest conversation with PI about IDP) and 22.4% (found IDP helpful) may be misleading to casual readers because these percentages include respondents who didn’t complete an IDP at all—and as reviewer 5 points out, they would have little basis for judgement. While the authors do report the value of 38.4% (% of those who completed an IDP and found it helpful), evidence that the aforementioned percentages can be misleading is found in the reviews of this manuscript, with the “22.4% value” cited as evidence that IDPs may not be effective strategies for improving postdoc outcomes. I recommend removing these two percentages from the graph and, within the text, reporting/highlighting the responses from only those who completed an IDP—which is in line with the rest of the analysis in the manuscript.
2. The authors emphasize that the IDP’s value has decreased to less than 40%, specifically comparing their results to Hobin *et al. *2014 ^1^. They addressed our concern somewhat and indicate later in the manuscript that care should be taken in comparing this current manuscript to other work. However, this point should be made (and further clarified) alongside their comparison because the study designs are quite different—both in the: a)  number & type of questions on IDP effectiveness & b) the manner in which the questions are asked. The Hobin *et al. *paper asks a series of several questions specifically addressing the helpfulness of different aspects of IDPs (ex: in identifying careers, assessing skills, facilitating communication, etc.), and then asks “How helpful was the IDP process overall?” In this study, one question is asked about IDP effectiveness—individuals are asked about their level of agreement with the statement:  “I find the IDP process helpful to my career development.”  Since it is emphasized that IDP effectiveness has decreased (a key result in this manuscript), readers could better compare these outcomes if a description about the nature & degree of difference between these study designs is included.
3. In line with other reviewers’ concerns about Figure 2’s clarity, I feel that it is important to reword Figure 2’s title & legend in order to clearly explain what this graph is showing, as it is not readily intuitive to readers. Likewise, some of the manuscript text should be clarified to remove ambiguity—RE: “For example, 62.2% of those respondents who indicated that they could have an honest conversation with their PI/advisor found that the IDP process was helpful to their career versus 26.3% of those who disagreed.” *...suggest to change to something like:* “For example, 62.2% of those respondents who indicated that they could have an honest conversation with their PI/advisor found that the IDP process was helpful to their career versus 26.3% of those who still found the IDP process helpful but disagreed that they could have an honest conversation.”  
4. Regarding the issue of ‘additional context’ – as this current study specifically addresses effectiveness of ‘individual development plans’ among postdocs— it will be helpful to readers to elaborate a bit more on the related work mentioned previously (Davis 2006 ^2^). The Davis study identifies many positive ‘correlates of success’ associated with postdocs who have a written training plan—although the plans referred to by Davis were not specifically termed ‘IDPs’—in principle, the idea of a written training plan (among postdocs) and associated outcomes is highly related/relevant to this current work.

I have read this submission. I believe that I have an appropriate level of expertise to confirm that it is of an acceptable scientific standard, however I have significant reservations, as outlined above.

## References

[1] HobinJACliffordPSDunnBMRichSJustementLB: Putting PhDs to work: career planning for today's scientist. *CBE Life Sci Educ*.2014;13(1) : 10.1187/cbe-13-04-0085 49-53 10.1187/cbe-13-04-0085 24591503PMC3940462

[2] Geoff Davis: Improving the Postdoctoral Experience: An Empirical Approach. *Science and Engineering Careers in the United States: An Analysis ofMarkets and Employment*.2009;99-127 Reference source

# Peer review: Referee response for version 2

**DOI:** 10.5256/f1000research.18327.r39878

**Published:** 2018-11-12

**Authors:** Kristen L. W. Walton

**Affiliations:** 1 Department of Biology , Missouri Western State University, St. Joseph, MO, USA

The authors have addressed my comments in the initial review.  I have no further concerns or suggestions for improvement.

I have read this submission. I believe that I have an appropriate level of expertise to confirm that it is of an acceptable scientific standard.

# Peer review: Referee response for version 2

**DOI:** 10.5256/f1000research.18327.r39880

**Published:** 2018-11-08

**Authors:** Adriana Bankston

**Affiliations:** 1 The Future of Research, Inc., Abington, MA, USA

The authors have addressed my concerns. I appreciated the additional references and explanations to some of the points previously raised, including better contextualizing of this work in prior literature, as well as the comparisons between the use and effectiveness of the IDP between postdocs and PhD students. I do think additional studies on this topic will be helpful in order to appropriately train various populations of researchers. 

 The edits to the figures indicated (Figure 2 and Supplementary File 3) are also very helpful to clarify the analyses made in particular for the Likert scale data. I would have been curious to see how race, ethnicity, U.S. citizenship, and other variables in Supplementary File 3 affect the responses to the IDP process, but that may not be possible with this sample size. Overall, I think these edits significantly improved the publication.

I have read this submission. I believe that I have an appropriate level of expertise to confirm that it is of an acceptable scientific standard.

# Peer review: Referee response for version 2

**DOI:** 10.5256/f1000research.18327.r39879

**Published:** 2018-11-07

**Authors:** Richard McGee

**Affiliations:** 1 Feinberg School of Medicine, Faculty Affairs, Northwestern University, Chicago, IL, USA

I feel the authors have done a good job of addressing my concerns and questions.

I have read this submission. I believe that I have an appropriate level of expertise to confirm that it is of an acceptable scientific standard.

# Peer review: Referee response for version 2

**DOI:** 10.5256/f1000research.18327.r39881

**Published:** 2018-11-06

**Authors:** Jonathan S. Wiest, Chanelle Case Borden

**Affiliations:** 1 National Cancer Institute (NCI) , Rockville, MD, USA; 2 National Cancer Institute (NCI), Rockville, MD, USA

Thank the authors for their revisions, the clarity of the article has increased.  We have some additional thoughts regarding our initial review:

1. Our first point was clarified. Thank you.
2. However, the source of the 38.4% is still unclear. The author’s now make clear they are only including the 99 people who completed IDPs. However, 41 people (out of the total 183 people) say that it is useful, which would be 41%. The corrected heading in Supplemental Figure 3 now states the percentage, but does not include the N.
3. We still wonder if the IDP effectiveness is clearly stated/defined. The data (e.g. the numbers) are clearer, however, the definition of effectiveness could be clearer. The questions that respondents are asked are about “helpfulness,” and effectiveness is inferred. We agree with Reviewer 2, in that the authors should provide additional context from the literature regarding this aspect. My recommendation would be to further parse out the two issues: 1) effectiveness alone (e.g. career preparation) and 2) effectiveness and mentor relationships.

We have read this submission. We believe that we have an appropriate level of expertise to confirm that it is of an acceptable scientific standard, however we have significant reservations, as outlined above.

# Response

**Published:** 2018-11-06

**Authors:** Nathan Vanderford

**Affiliations:** University of Kentucky, USA

Dear Drs. Wiest and Case Borden,

 Thank you for this new review.

 To clarify your second point, the 38.4% was derived from only those respondents that completed an IDP (N = 99) AND indicated that it was helpful to their career development (N = 38); 38/99 = 38.4%.

 As for your third point, as stated in the response to other reviewers’ comments, some of our survey questions were ambiguous, thus making the interpretation a bit difficult. Additionally, other than the Hobin et al. article from 2014 and our recent work on the use and effectiveness of the IDP in graduate students, we are unaware of other literature that specifically addresses the use and effectiveness of the IDP. Given that this in an understudied area, it is difficult to put our work into additional context. Importantly, however, we believe that the lessons learned (positive and negative) from this study will be informative to future work in this area.

 Sincerely,

 Nathan L. Vanderford

# Peer review: Referee response for version 1

**DOI:** 10.5256/f1000research.17029.r37949

**Published:** 2018-09-18

**Authors:** Richard McGee

**Affiliations:** 1 Feinberg School of Medicine, Faculty Affairs, Northwestern University, Chicago, IL, USA

As noted by Review 1, the manuscript is well written and easy to follow. However, I would agree with both of the other reviews that problems arise from both the survey questions and decisions on method of analysis. The first 3 questions about completing an IDP don’t really lead to a clean number of how many people do and do not complete one. A person could answer no to being required to complete an IDP but could be doing it voluntarily.  And the issue of doing it at all vs. doing it annually makes the numbers even more ambiguous and difficult to sort out.  An additional challenge comes from the 5 choices for questions 1-3 when they are really only yes/no situations. It is hard to imagine what could lead a person to be neutral on these 3 questions.

 I would also agree with the other reviewers that there is no rational for combing neutral responses with negative responses. This will skew the interpretation to a negative side without any evidence the respondent meant it to.  This is one reason surveys often don’t provide the neutral point as the data are very difficult to interpret. At least, as reviewer 2 points out, including neutral as a distinct option would allow readers to reach their own conclusions.

 I also agree that % responses to questions 4 and 5 should be based only on those who actually complete the IDP as others really don’t have any basis for judgement. But as noted above and by other reviewers, this number of those who completed it is illusive from the question designs.

 RE: Figure 2, as pointed out by the other reviewers, it really does not reveal effectiveness of the IDP.  At most it displays associations between some of the questions. I also would raise a concern with the 2 questions about mentors: “My PI/advisor provides real mentorship” and “My PI/advisor provides ample support”. Both of these are very ambiguous – e.g. is the intent to separate ‘real’ mentorship from some other form of mentorship? And what kind of ‘support’ – financial, psychosocial, professional? This level of ambiguity adds to concerns for including neutral responses with the disagree categories because neutral could easily reflect not knowing what the questions are asking for.

I have read this submission. I believe that I have an appropriate level of expertise to confirm that it is of an acceptable scientific standard, however I have significant reservations, as outlined above.

# Response

**Published:** 2018-10-22

**Authors:** Nathan Vanderford

**Affiliations:** University of Kentucky, USA

Dear Dr. McGee,

 We thank you for your review, which has guided our revisions. We respond to your major comments below.

 In retrospect, we agree with your comments regarding our survey questions related to discerning a number and percentage of respondents who completed an IDP. Looking back, we could have asked a simple yes/no question(s). That said, what we have done in our analysis is to use the unique responses to questions 2 and 3 within our survey (please refer to the survey instrument within Supplemental File 4) to arrive at the number and percentage of respondents that completed an IDP. Out of the 112 respondents that agreed to both survey questions 2 and 3, 13 agreed to both questions and these respondents were subtracted from the total to obtain the number and percentage of  total respondents who completed an IDP (112 – 13 = 99; 99/183 = 54.1%). While not as clear-cut as a simple yes/no question, we are confident that this approach allows us to discern the number/percentage of our respondents who completed an IDP.

 We also agree with you and several other reviewers regarding the analysis of the Likert data. We have now split out the neutral responses as an independent category and we have updated the text, data, figures, and files accordingly.

 We apologize for the lack of clarity regarding the analysis of variables that associate with IDP effectiveness with respect to analyzing only those respondents that completed an IDP. In fact, this is how we designed the analysis and we have clarified this in the text.

 Figure 2 does indeed show variables that correlate with IDP effectiveness. We measured IDP effectiveness by asking respondents the question “I Find the IDP Process Helpful to my Career Development” and then we used this as an outcome variable to understand if variables such as mentorship associate with IDP effectiveness. Again, we have clarified this in the new version of the article.

 We agree that there are levels of ambiguity in some of our questions. This was, in some cases, by design. In hindsight, however, we could have clarified some of the questions. That said, we believe that the lessons learned from our study design (including the survey design) and our data/findings will be informative to future studies that look to better understand the use and effectiveness of the IDP.

 Thank you again for your critique. We believe that your comments and those of the other reviewers have improved our work. We look forward to your next review.

 Sincerely,

 Nathan L. Vanderford

# Peer review: Referee response for version 1

**DOI:** 10.5256/f1000research.17029.r37951

**Published:** 2018-09-18

**Authors:** Kristen L. W. Walton

**Affiliations:** 1 Department of Biology , Missouri Western State University, St. Joseph, MO, USA

This article summarizes data from a subgroup of individuals surveyed about their use of an Individual Development Plan and other factors. Data on the effectiveness and usefulness of the IDP is important to justify policies that require postdoctoral scholars and PhD students to complete an IDP. The findings are interesting and concisely presented. The authors appropriately acknowledge several limitations to this survey. Overall, this manuscript adds important data to a field that is very difficult to quantify, given the variability in the IDP across institutions and training programs. I do have some questions and suggestions to strengthen this manuscript:

1.  As noted by other reviewers, the separation of the Likert scale data into “agree” and “disagree” categories, with “neutral” included in the “disagree” category, has the potential to skew results towards the “disagree” category.  It would be helpful to analyze the data with neutral responses listed as a separate category. 
2. The survey population demographics as reported in Supplementary File 2 show that the population of respondents was 80.7% white. How do the demographics of the survey population reflect the national postdoc population demographics? The numbers in some categories are likely too small to analyze in a statistically meaningful way, but it would be interesting to determine whether different demographic groups (race, gender, etc) had similar responses regarding the usefulness of the IDP and/or mentoring relationships. 
3.  I agree with other reviewers that this paper has relatively minimal introduction and discussion to place it in the context of other work. The issues faced by postdocs are not identical to those faced by PhD students, and there are multiple recent publications discussing the problems facing postdocs (for example, The Postdoc Experience Revisited, *National Academies Press* 2014 ^1^; Alberts et al, PNAS 2014 ^2^). The data in this manuscript that show that only 22.4% of survey respondents felt that the IDP process was helpful to career development suggest that this process may not be an effective strategy for improving the postdoctoral experience and outcomes.

I have read this submission. I believe that I have an appropriate level of expertise to confirm that it is of an acceptable scientific standard, however I have significant reservations, as outlined above.

## References

[1] National Academy of Sciences: The Postdoctoral Experience Revisited. *The National Academies Press*.2014; https://doi.org/10.17226/18982 10.17226/18982 25590106

[2] AlbertsBKirschnerMWTilghmanSVarmusH: Rescuing US biomedical research from its systemic flaws. *Proc Natl Acad Sci U S A*.2014;111(16) : 10.1073/pnas.1404402111 5773-7 10.1073/pnas.1404402111 24733905PMC4000813

# Response

**Published:** 2018-10-22

**Authors:** Nathan Vanderford

**Affiliations:** University of Kentucky, USA

Dear Dr. Walton,

 Thank you for your review of our article. We have responded to your major concerns one-by-one below.

 As mentioned in several of the other responses to reviewers’ comments, we have reanalyzed our data using the neutral responses as a separate category. All the text, data, figures, and files have been updated accordingly.

 We did collect data on the race and ethnicity of our respondents and we observed no significant differences between the groups regarding their response to whether they found the IDP helpful to their career development. We agree that our sample size may be too low to definitively draw any hard conclusions in this regard, however. It will be interesting to re-visit this question with a much larger sample size. One could envision differences given what is known about minority populations and the training and career outcome pipelines. Understanding these differences is critical in order to develop interventions that can fit the needs of specific populations.

 We have added a bit of additional text and literature to further contextualize our work. Of note, however, this article type has a 1,000 word limit and thus there is limited space to house a comprehensive literature review on all the related trainee career development topics. As such, we have focused on discussing the pertinent IDP literature.

 Thank you again for your time and review. We look forward to your second review.

 Sincerely,

 Nathan L. Vanderford

# Peer review: Referee response for version 1

**DOI:** 10.5256/f1000research.17029.r37952

**Published:** 2018-09-14

**Authors:** Adriana Bankston

**Affiliations:** 1 The Future of Research, Inc., Abington, MA, USA

**General comments:**

 This publication addresses the very important topic of use and effectiveness of the individual development plan (IDP) for trainees. This is not a trivial topic to address and I commend the authors for this analysis in the context of current literature. I also appreciated the valuable insights on how the effectiveness of the tool is associated with positive mentoring, pointing out the need for strong relationships with advisors that can affect career trajectories for graduate students and postdocs.

**General considerations:**

 On the background side, not being terribly familiar with the already existing literature on this topic, I would recommend a bit more description of prior studies that could frame this work, and its novelty in the context of existing literature. However, I recognize that it’s also possible literature on the effectiveness of the IDP may be limited, in particular given the abstract nature of this concept. Without having read more background, but judging from the information presented in the introduction, my immediate impression is that prior studies looked at PhD students whereas this work examines postdoctoral researchers. Moreover, the authors mentioned that this was a multi-institutional analysis, which leaves me wondering whether the novelty of this work is looking at a different population (postdocs vs. graduate students) or whether it is related to the number or type of institutions that prior studies hadn’t examined in this context.

 On the technical side, the abstract mentions that the authors looked at data from 183 postdocs, although from the methods section it appears that both graduate students and postdocs were examined for a period of March to June 2016. Given that a study of 663 PhD students was previously performed as referenced in the introduction, it would be helpful to see an articulation of the novelty that this current study brings over previous work. It would also be beneficial to clarify whether both populations were included in all the analyses in this publication (in particular figure 2, which appears to refer to PhD students, whereas figure 1 refers to postdocs), and to expand more on the similarities and differences between the effectiveness of the IDP on these two populations. While this is mentioned in the discussion section, if both graduate students and postdocs were analyzed more in-depth in these studies (also taking into account other aspects of the data in this publication), it would make for an interesting comparison as to whether or not the IDP has more of an effect on the career trajectories of one group or another. And while positive mentoring is required for both populations, postdocs may already be well on their way towards a more obvious career path than PhD students are. While the authors state similar trends in these topics, it would be helpful to take this analysis a step further and determine how IDP effectiveness affects career choices for these groups.

 In terms of data analysis, the authors state that they grouped together the agree/strongly agree, and neutral/disagree/strongly disagree responses. I wondered why this is the case, perhaps it could be a low sample size that may not enable meaningful conclusions. However, in order to gain a fully comprehensive picture of the issue at hand, I would recommend displaying and analyzing data from each of these categories separately. I believe that for such a topic that is difficult to quantify, it will be important to dissect the prevalence of each of these responses. Examining the various categories (availability of programs, attendance and usefulness) in Supplementary Data 2 could be utilized for a more thorough analysis of this topic. While I wonder how usefulness can be assessed in a practical sense, it was also disheartening to see the percentage of respondents who indicated that they do not attend or did not find available programs helpful. Perhaps this is an area that should be further explored in terms of which programs would likely be helpful for trainees to have. It would also be interesting to dissect further the correlation between the usefulness of the IDP and subsequent career paths chosen. For example, did individuals who found the IDP helpful end up in the top career path predicted by the IDP, and are they currently satisfied in their position? If so, this might indicate that the IDP was useful in helping them achieve desired career goals. The IDP could also open them up to career paths they hadn’t considered before, which would demonstrate the added value of this tool for training and career development.

 I appreciated the data transparency in this publication (for example in Dataset 1, Supplementary Data 4). Given the wealth of information and number of questions asks, further analyses of these existing data looking at the effect of other variables on IDP effectiveness would provide a thorough analysis of how we might improve the IDP process based on barriers faced by particular groups. I would also suggest detailing the data analysis and quantification procedure used in this publication (as opposed to referencing prior publications with the information), in order to clarify how percentages in the results were calculated.

 I also wonder whether it’s possible to examine other variables together to make predictions that would enrich this publication in the future. For example, how do factors such as race, ethnicity, U.S. citizenship, and others, affect the responses to the IDP process (Supplementary Data 3). While these may not have been the primary objectives of the authors, this type of analysis would add another layer of complexity to whether the IDP is useful to various groups, whose career decisions may also be affected by additional factors. This publication does contain a large amount of raw data that I think could be utilized for a more thorough analysis of how various factors contribute to the effectiveness of the IDP. However, given the data is self-reported and there may be a limited number of responses in each of these categories, it may be difficult to assess the effect of such variables with the current dataset.

**Feedback on results:**

 It was somewhat disheartening to see the percentage of postdocs who had completed the IDP without discussing it with their advisor (Figure 1), and the percentage of those that had honest conversations with their advisors was also not terribly high. These factors point to barriers towards positive mentoring relationships in academe, as well as obstacles to career development for trainees. They could affect the ability of trainees to follow desired career paths, or having to prepare for transitioning into these careers without their advisors knowing, especially if the advisor does not approve of their non-academic career choice. This fundamentally points to systemic flaws in academe and how the enterprise needs to change in order to better support trainees who are using the IDP as a guide to explore career options. Importantly, this also requires advisors to point trainees in the right direction, and be a sounding board during career transitions. I also wonder whether there is a connection between the lack of discussions with the advisor and their ability to have honest conversations (Figure 1), as it appears that this could be a layered response (i.e. they were either having or not having these conversations, and if they were, how honest did the trainees feel they could be with their advisors in terms of desired career options?). I think drawing a connection between these two variables could be valuable to investigate in terms of the barriers affecting the ability of trainees to pursue various career paths, and assess the usefulness of the IDP process for these particular careers.

 Figure 2 somewhat addresses this concern, and it was valuable to see that positive mentoring and having honest conversations with advisors can influence the responses of trainees on IDP effectiveness. There is a lot of really valuable information in this figure in terms of how we can improve faculty training to be more supportive of the career choices of their trainees, so that they feel valued and prepared for taking on other careers besides academia. Given the importance of these factors, it would also be interesting in the future to look at how positive relationships with advisors affect other aspects of training and career preparation for trainees. While factors such as the advisor being an asset to their career, providing ample support and positively impacting their emotional and mental well-being, among others variables, are likely very difficult to assess, I believe these are critical investigations that should be pursued further and more in-depth to better understand how to train the next generation of researchers. Along the lines of these ideas, putting these findings into a larger context would be really helpful in discussing how to better equip faculty to help trainees be successful in their desired careers.

**Additional recommendations:**

 It was interesting to learn about the comparison between IDP use and effectiveness for PhD students and postdocs, as detailed in the discussion section. I was surprised to see that postdocs weren’t required to complete the IDP to the same extent that PhD students were, did not discuss the results of completed IDPs with their advisor as much, and found the IDP less helpful for their career development. This observation that merits further investigation, as to whether the lack of usefulness of the IDP for postdocs was due to their inability to discuss it with their advisors, or whether other factors were also involved. I would also be curious to know more about why there is a lesser requirement for postdocs to complete the IDP, and whether reversing this trend would result in a greater percentage of postdocs actually pursuing desired career paths as opposed to traditional academic routes.

 In terms of comparing data from PhD students and postdocs, I wonder whether these surveys and subsequent analyses were performed on both populations at the same time (during March to June 2016 as described in the methods) or whether the data discussed here on PhD students came from a previous publication. This analysis could also provide insights into whether we should target certain populations more in terms of IDP assessments, and which populations within academe the IDP is likely to be more useful for in terms of career exploration. For a more extensive analysis, it would also be interesting to compare all of the aspects in Figure 2 between PhDs and postdocs, in order to determine the effect of mentoring relationships on career trajectories of trainees at various stages in their careers.

 I appreciated that the authors pointed out limitations of the study in the discussion section, including as it relates to institutional variability. Indeed, Supplementary File 1 indicates that there are very few individuals at the institutions shown in the dataset, and many are at missing institutions. I imagine there is also quite a variability between these institutions in terms of size, number of postdocs, and the type of career development opportunities available that could supplement the IDP findings for trainees. These variables could influence how trainees rate the usefulness of the IDP, in terms of whether additional resources exist for them to further explore careers that were indicated as a good fit from the IDP. For example, it is possible that a limited knowledge on available career options, either due to the lack of resources or the inability of their advisor to help (in addition to not being able to find another suitable mentor to assist with career exploration), trainees may rate the usefulness of the IDP as lower than those with more external information available.

 Increasing the sample size of respondents from each institution would also provide a clearer picture of how institutional environments affect career trajectories for trainees. In addition, incorporating other variables into the evaluation of institutions would enable various types of comparisons to be made about IDP effectiveness by trainees from diverse backgrounds, or those in institutions of a certain size or geographical area. These are also factors that could affect their career development - for example a larger city might offer opportunities to interact with other postdocs and take advantage of multi-institutional career development opportunities, which trainees in other geographical areas may not have access to.

**Broader picture comments:**

 I agree with the authors that faculty should receive mentorship training and it would be helpful to see further elaboration by the authors on how this could be achieved. For example, mentorship training for faculty could include manuals with both internal and external resources and contacts from various career paths that trainees might want to pursue, thus enabling them to better train their postdocs for appropriate careers. There is also currently the barrier of trainees not being able to have honest conversations with advisors about their career options, therefore faculty attitudes need to change in order to allow postdocs to pursue non-academic careers.

 I also agree that a better career development infrastructure is needed, and that this would be a massive undertaking. Incorporating the findings from this publication, however, into current literature on these topics (and efforts made by others to reform career training in universities) would be a helpful beginning to understanding how such an infrastructure could be developed. Implementing the IDP as a mandatory training for postdocs at the bench as part of their annual assessment may already be happening at some universities, however we should also keep in mind that IDPs are really only the beginning of the career development process - while it is the responsibility of trainees to utilize their IDP results for further career exploration, an infrastructure that supports this process is imperative to their success. This infrastructure I envision could be internal to the university, or there could be an external entity developing resources for several universities to utilize for helping trainees explore career options. As part of this work, the authors could also consider developing a rubric to measure IDP effectiveness as it relates to their ability to achieve career goals outlined in the IDP. I would also be curious to see a rubric for assessing other factors that can influence this effectiveness (such as those in Figure 2) and trying to understand more about particular elements that go into each of these factors.   

 Overall, this is a well written manuscript tackling an issue that is difficult to quantify but very important to study from the context of training the next generation of scientists. I believe that more of an in-depth literature overview, further analysis of the existing data and collection of additional data, and a more extensive discussion of the recommendations for change around faculty training to support postdocs, would greatly strengthen the manuscript in the future. I believe these findings are a valuable foundational start to these questions, conducting further investigations on this topic can provide more in-depth understanding of the potential that the IDP could have for training graduate students and postdocs for being successful in their chosen careers.

I have read this submission. I believe that I have an appropriate level of expertise to confirm that it is of an acceptable scientific standard, however I have significant reservations, as outlined above.

# Response

**Published:** 2018-10-22

**Authors:** Nathan Vanderford

**Affiliations:** University of Kentucky, USA

Dear Dr. Bankston,

 Thank you for your very extensive report. We have responded to your major critiques/comments below.

 We have added a bit more text and references to better contextualize our work.

 We apologize for the confusion over the study population. In this work, we report only on data obtained from postdoctoral trainees. Our previous report was focused on IDP use and effectiveness in PhD students. These data were collected at the same time, but we choose to analyze the postdoctoral trainee and PhD student data separately.

 As mentioned in several of the responses to other reviewers’ comments, we have now broken out the neutral responses to our Likert scale questions and we now present these data separately. All the applicable text, data, figures and supplemental files have been updated accordingly.

 Much of your additional comments focus on additional data analysis and comparisons of the postdoctoral trainee and PhD student data. We agree that your suggestions are very important and you have posed very interesting and essential questions. We, however, feel that your suggestions are out of scope for the current study. This study was submitted as a short Research Note (which has a 1,000 word limit). These article types are meant to convey findings that can be described in a short report. One of our goals of this work was to obtain preliminary findings that can inform other work on the use and effectiveness of the IDP. Additional IDP use and effectiveness data that should be collected with a revised survey instrument that is informed by our work will allow for such additional analyses in the future. 

 Thank you for your time and comprehensive report. We hope that you will favorably consider our revisions in light of our changes that address your major critiques and those of the other reviewers. We look forward to reading your next review.

 Sincerely,

 Nathan L. Vanderford

# Peer review: Referee response for version 1

**DOI:** 10.5256/f1000research.17029.r36897

**Published:** 2018-08-30

**Authors:** Tammy Collins

**Affiliations:** 1 Office of Fellows' Career Development, National Institute of Environmental Health Sciences, National Institutes of Health, Research Triangle Park, NC, USA

The authors report on the usage of IDPs by postdoctoral scholars, which is both a timely and fundamental topic within the broader graduate and postdoctoral professional development community. This work extends beyond the authors’ recently published article on IDP usage among doctoral students ^1^ to showcase how the instrument is currently being used by postdocs. The aforementioned manuscript on doctoral IDP usage extensively discusses policy and other issues surrounding IDPs, while this manuscript is lean on discussion. It would therefore benefit from including prior literature on correlates of success associated with postdocs who have written plans—which would have the added benefit of placing this work into a broader context (for example, see: Davis 2009 ^2^).

 There are a number of points that should be addressed, and they are outlined as follows:

1. The survey instrument asks questions on a 5-point Likert scale of strongly agree, agree, neutral (neither agree nor disagree), disagree, & strongly disagree. However, when analyzing the data, the authors report percentages either as ‘does not agree’ (also reported as ‘disagree’) or ‘agree’ - with ‘strongly disagree, disagree & neutral’ all grouped together as a ‘does not agree’ response. It seems that lumping “neutral (neither agree nor disagree)” into the ‘does not agree’ category would skew results (both in this manuscript and the manuscript on doctoral IDP usage) towards ‘does not agree.’ It is recommended that the authors reanalyze the data and report the ‘neutral’ responses as a third category in order to more accurately reflect intended answers/percentages.
2. I agree with reviewer 1 that it is unclear how the authors calculated that 54% completed an IDP; like reviewer 1, I also calculated that 61% completed an IDP either with or without their PI. Similarly, I also agree with reviewer 1 that it is unclear where the 38.4% value came from (percent of respondents that completed an IDP who found the tool helpful). Furthermore, in figure 1, a value of 22.4% of all respondents (whether they complete an IDP or not) is listed as saying the IDP process is helpful to their career development. Since “22.4%” also includes those who never completed an IDP, reporting it in this manner seems to bias perceptions of the tool as unhelpful.
3. Aside from the unclear derivation of percentages discussed in point #2, the questions in the survey instrument do not seem to allow confident discernment of who actually completed an IDP. For example, respondents who disagree with the question “I complete an IDP annually with my PI/advisor” could have actually completed an IDP with their PI at the beginning of their postdoc and/or possibly in year 3 of their postdoc—how much weight were respondents giving to the word ‘annually’? Another question reads: “I complete an IDP but I do not discuss it with my PI/advisor” - could respondents complete two different IDPs - one with and one without their PI (and thus agree to both the former and latter questions)? 
4. The authors assert that the usefulness of an IDP has decreased since 2014, specifically comparing their results to that in (Hobin *et al. *2014 ^3^). However, in the (Hobin *et al. *2014) paper, an IDP’s overall value is reported either as ‘not helpful’ ‘neutral’ or ‘helpful’. It seems that the results in this manuscript could be compared more accurately with the (Hobin *et al. *2014) data by showing the ‘neutral’ responses, rather than lumping them with ‘does not agree’ (see point 1 above). Additionally, it would be helpful to point out the limitations of comparing these two studies (ex: address key differences between the two survey instruments regarding how the IDP questions were asked (and how this might bias responses), address potential respondent audience differences, etc.). 
5. Like reviewer 1, I also feel that there are limited questions that address what one might consider IDP ‘effectiveness.’ The manuscript would thus be strengthened by discussing IDP indicators that have been previously reported in the literature (such as measures outlined by (Hobin *et al. *2014) - ex: the value of an IDP in helping with self-assessment, helping identify career paths, helping identify skills to strengthen, etc.). Furthermore, (Davis 2009 ^2^) reports an in-depth analysis of results from a Sigma Xi Postdoc Survey - identifying many positive correlates of success associated with postdocs who develop a written plan at the outset of their careers (ex: higher publication rate, grant submission rate, better supervisor relationships, etc.). Since the main point of this manuscript is to discuss the use and effectiveness of IDPs among postdocs, it would benefit from elaborating upon such postdoc-specific contextual literature - as well as other literature that documents the general benefits of goal-setting, which is a primary function of IDPs (Locke *et al. *2002 ^4^).
6. (Minor) The authors indicate that “additional research is needed,” and it would thus be beneficial to clarify the research questions that should be addressed. For example, it seems that it would be useful to determine the effects of various parameters on IDP effectiveness such as: 1)  when in training an IDP is completed; 2) inclusion/ exclusion of IDP components (such as self-assessment, career exploration, skill-building, goal-setting, etc.); 3) prior experience with/completion of an IDP as a PhD student; 4) completing an IDP of their own accord versus doing so because it is required; 5) receiving training on how to create/implement an IDP; 6)  using a specific IDP instrument (ex: myIDP) versus an institutionally-developed IDP, etc..
7. (Minor) As an additional point - since ‘IDP effectiveness’ is subjective as the authors point out, perhaps future studies should address better-defining these parameters so that common IDP evaluation methods can be adopted within the broader community. It would also be especially helpful to ascertain **what** IDP tools are being used, and **how** they are being implemented so that standard ‘correlates of effectiveness’ could be tied to specific IDP instruments (or components) and the manner in which they are employed.

I have read this submission. I believe that I have an appropriate level of expertise to confirm that it is of an acceptable scientific standard, however I have significant reservations, as outlined above.

## References

[1] VanderfordNLEvansTMWeissLTBiraLBeltran-GastelumJ: A cross-sectional study of the use and effectiveness of the Individual Development Plan among doctoral students. *F1000Res*.2018;7: 10.12688/f1000research.15154.2 722 10.12688/f1000research.15154.2 30026933PMC6039936

[2] DavisG: Improving the postdoctoral experience: An empirical approach. *Science and engineering careers in the United States: An analysis of markets and employment*.2009;99-127

[3] HobinJACliffordPSDunnBMRichSJustementLB: Putting PhDs to work: career planning for today's scientist. *CBE Life Sci Educ*.2014;13(1) : 10.1187/cbe-13-04-0085 49-53 10.1187/cbe-13-04-0085 24591503PMC3940462

[4] LockeELathamG: Building a practically useful theory of goal setting and task motivation: A 35-year odyssey. *American Psychologist*.2002;57(9) : 10.1037/0003-066X.57.9.705 705-717 10.1037/0003-066X.57.9.705 12237980

# Response

**Published:** 2018-10-22

**Authors:** Nathan Vanderford

**Affiliations:** University of Kentucky, USA

Dear Dr. Collins,

 Thank you for your review. Your comments and critique have been very helpful in guiding our revisions. We respond to your major points below.  

 We appreciate your comments and concerns regarding our decision to combine the neutral Likert scale responses with the disagree and strongly disagree responses. As such, we have now split these responses out and present three categories (agree, neutral, and disagree) in our analysis. The applicable dataset, figures, supplemental files, and text have been revised accordingly.

 As mentioned in our response to Drs. Wiest and Case Borden, we sincerely apologize for the confusion over our reporting of the percent respondents to several questions. In particular, we analyzed the unique responses to questions 2 and 3 in our survey (please refer to the survey instrument within Supplemental File 4) to arrive at both the number and the percentage of respondents that had completed an IDP. Out of the 112 respondents that agreed to both survey questions 2 and 3, 13 agreed to both questions and these respondents were subtracted from the total to obtain the number and percentage of  total respondents who completed an IDP (112 – 13 = 99; 99/183 = 54.1%). Supplemental File 2 reports on the data from all respondents while the univariate association analysis shown in Supplemental File 3 reports on the data only from the 99 unique respondents that reported completing an IDP. We have clarified this in the new version of the article. It is important to note that the frequency data in Supplemental File 2 is not additive because of the 13 respondents who agreed to both questions 2 and 3.

 In retrospect, we agree that our questions can make it difficult to discern which respondents uniquely completed an IDP. In hindsight, we could have asked a simple yes/no question about whether trainees had completed an IDP. That said, we are confident that our method of combining and de-duplicating the responses to questions 2 and 3 allow us to determine which of our respondents have completed an IDP.

 The new analysis of our Likert scale data allows us to more clearly and directly compare our results to the 2014 Hobin et al. data. Additionally, we have also specified in our limitations section that care should be taken in making such comparisons because of the analysis of different populations (e.g., although both populations were postdocs, there could be institutional differences, etc.) and different study designs.

 We have added a bit of additional text and references to better contextualize our work, to further clarify some limitations of the study, and to better define future research questions. Of note, however, this article was submitted as a short Research Note article type which has a 1,000 word limit and thus space is limited regarding adding a comprehensive literature review related to PhD trainee career development. We have thus focused on the IDP-related literature.

 Thank you again for your review. We look forward to your second review in light of our revisions. We feel that your comments and that of the other reviewers have strengthened the article.  

 Sincerely,

 Nathan L. Vanderford

# Peer review: Referee response for version 1

**DOI:** 10.5256/f1000research.17029.r36439

**Published:** 2018-08-09

**Authors:** Jonathan S. Wiest, Chanelle Case Borden

**Affiliations:** 1 National Cancer Institute (NCI) , Rockville, MD, USA; 2 National Cancer Institute (NCI), Rockville, MD, USA

In this study, the authors seek to determine the effectiveness of utilizing an IDP during the training stage of a biomedical career. Using survey data from a larger online study of overall health and well-being, 183 trainees responded to questions regarding the IDP using the Likert scale. While 45.4% of the respondents were required to complete an IDP, in total, 54.1% completed an IDP (with or without their mentor’s support).  Only of third of those who completed the IDP, however, found the tool useful for career development. Most notably, the authors found positive correlation between positive mentoring and the effectiveness of utilizing an IDP. 

 Overall, this is a well written manuscript addressing an important topic in the training community. However, there are several points that this reviewer found to be confusing.

 First, the authors state that 54.1% of respondents completed an IDP, however, based on the data in Figure 1 and Supplementary File 2, 112 respondents (61%) of respondents completed an IDP.  Further, in Figure 2, the authors are basing their conclusions on 99 respondents.  While this is 54% of the 183 respondents, it is unclear why the total of 99 was used as opposed to the 112. 

 Second, the authors state that 38.4% of respondents who completed an IDP found the tool to be useful to their career development and reference Supplementary File 3. Yet, upon closer inspection, a question about IDP usefulness is missing from that document altogether. Thus, it is unclear where that percentage was derived.  The data in Supplemental File 2 shows that 22.4% of the total population found the tool effective, however, that is reflective of the total population, and not those who completed the IDP. Taking those findings into consideration, the percentage of those who utilized the IDP and found it effective is 36.6%.

 Lastly, it is unclear how Figure 2 is an analysis of IDP effectiveness. To the authors credit, it is noted that effectiveness is subjective and the IDP structure can be a confounding factor. However, the questions in Figure 2 are more indicative of mentor effectiveness, and not that of the IDP. It is important to note that the respondents who have positive relationships with their mentors seem to be better prepared, but only half of those find the IDP effective.

We have read this submission. We believe that we have an appropriate level of expertise to confirm that it is of an acceptable scientific standard, however we have significant reservations, as outlined above.

# Response

**Published:** 2018-10-22

**Authors:** Nathan Vanderford

**Affiliations:** University of Kentucky, USA

Dear Drs. Wiest and Case Borden,

 Thank you for your review. Your critique has been very helpful as we have revised the article. Below we address the major issues you raised.

 We apologize for the confusion regarding the percent of respondents that completed an IDP. We analyzed the unique responses to questions 2 and 3 in our survey (please refer to the survey instrument within Supplemental File 4) to arrive at both the number and the percentage of respondents that had completed an IDP. Out of the 112 respondents that agreed to both survey questions 2 and 3, 13 agreed to both questions and these respondents were subtracted from the total to obtain the number and percentage of total respondents who completed an IDP (112 – 13 = 99; 99/183 = 54.1%). Supplemental File 2 reports on the data from all respondents while the univariate association analysis shown in Supplemental File 3 reports on the data only from the 99 unique respondents that reported completing an IDP. We have clarified this in the new version of the article.   

 We likewise apologize for the oversight of not listing the percentage (38.4%) of respondents that had used the IDP and found it useful to their career development in Supplemental File 3. We now include this data in the top portion of the table found in Supplemental File 3. It is important to note that this percentage (38.4%) is based only on those respondents that had completed an IDP (again based on unique respondents to questions 2 and 3 in the survey). The frequency data presented in Supplemental File 2 is not additive because of the 13 respondents who agreed to both questions 2 and 3. We have clarified this in the new version of the article. 

 Figure 2 does show a set of two category-level variables that are associated with IDP effectiveness. The asterisks in particular point to the significant differences in the proportions of the outcome (IDP effectiveness) among the levels of a given variable using the Chi-square test of proportions. We measured the outcome, IDP effectiveness, by asking respondents the question “I Find the IDP Process Helpful to my Career Development” Again, we have clarified this in the new version of the article.

 In closing, we have revised the article to address your comments as well as those of the other reviewers. We hope that you will favorably review the revised version of the article in light of our changes. Thank you again for your comments as we strongly feel that they have strengthened our work. We look forward to reading your next review.

 Sincerely,

 Nathan L. Vanderford
